# Mechanisms underlying the prolonged activation of the genioglossus following arousal from sleep

**DOI:** 10.1093/sleep/zsad202

**Published:** 2023-07-28

**Authors:** Andrew Dawson, Joanne Avraam, Christian L Nicholas, Amanda Kay, Therese Thornton, Nicole Feast, Monika D Fridgant, Fergal J O’Donoghue, John Trinder, Amy S Jordan

**Affiliations:** Melbourne School of Psychological Sciences, The University of Melbourne, Parkville, Victoria, Australia; Melbourne School of Psychological Sciences, The University of Melbourne, Parkville, Victoria, Australia; Department of Respiratory and Sleep Medicine and Institute for Breathing and Sleep, Austin Health, Heidelberg, Victoria, Australia; Melbourne School of Psychological Sciences, The University of Melbourne, Parkville, Victoria, Australia; Department of Respiratory and Sleep Medicine and Institute for Breathing and Sleep, Austin Health, Heidelberg, Victoria, Australia; Melbourne School of Psychological Sciences, The University of Melbourne, Parkville, Victoria, Australia; Melbourne School of Psychological Sciences, The University of Melbourne, Parkville, Victoria, Australia; Melbourne School of Psychological Sciences, The University of Melbourne, Parkville, Victoria, Australia; Melbourne School of Psychological Sciences, The University of Melbourne, Parkville, Victoria, Australia; Department of Respiratory and Sleep Medicine and Institute for Breathing and Sleep, Austin Health, Heidelberg, Victoria, Australia; Faculty of Medicine, The University of Melbourne, Parkville, Victoria, Australia; Melbourne School of Psychological Sciences, The University of Melbourne, Parkville, Victoria, Australia; Melbourne School of Psychological Sciences, The University of Melbourne, Parkville, Victoria, Australia; Department of Respiratory and Sleep Medicine and Institute for Breathing and Sleep, Austin Health, Heidelberg, Victoria, Australia

**Keywords:** obstructive sleep apnea, awakening, upper airway, pharyngeal dilator muscles

## Abstract

**Study Objectives:**

Transient arousal from sleep has been shown to elicit a prolonged increase in genioglossus muscle activity that persists following the return to sleep and which may protect against subsequent airway collapse. We hypothesized that this increased genioglossal activity following return to sleep after an arousal is due to persistent firing of inspiratory-modulated motor units (MUs) that are recruited during the arousal.

**Methods:**

Thirty-four healthy participants were studied overnight while wearing a nasal mask with pneumotachograph to measure ventilation and with 4 intramuscular genioglossus EMG electrodes. During stable N2 and N3 sleep, auditory tones were played to induce brief (3-15s) AASM arousals. Ventilation and genioglossus MUs were quantified before the tone, during the arousal and for 10 breaths after the return to sleep.

**Results:**

A total of 1089 auditory tones were played and gave rise to 239 MUs recorded across arousal and the return to sleep in 20 participants (aged 23 ± 4.2 years and BMI 22.5 ± 2.2 kg/m^2^). Ventilation was elevated above baseline during arousal and the first post-arousal breath (*p* < .001). Genioglossal activity was elevated for five breaths following the return to sleep, due to increased firing rate and recruitment of inspiratory modulated MUs, as well as a small increase in tonic MU firing frequency.

**Conclusions:**

The sustained increase in genioglossal activity that occurs on return to sleep after arousal is primarily a result of persistent activity of inspiratory-modulated MUs, with a slight contribution from tonic units. Harnessing genioglossal activation following arousal may potentially be useful for preventing obstructive respiratory events.

## Graphical Abstract

**Graphical Abstract:**
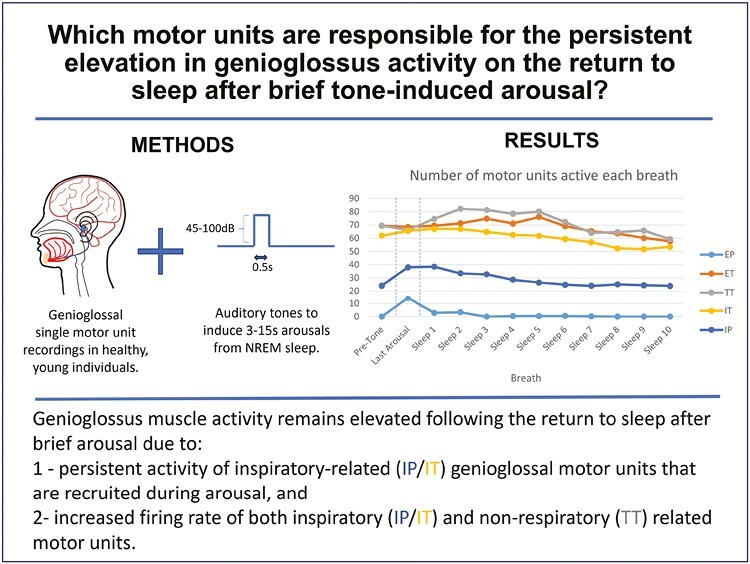

**Statement of Significance**

Arousals from sleep result in the activation of the largest airway dilator muscle (the genioglossus) that persists for 20-30s following the return to sleep. How genioglossus activity remains elevated following the return to sleep was unknown. This study documented the firing patterns and frequencies of genioglossus motor units (MUs) across arousal and the return to sleep and found that both recruitment of MUs with an inspiratory firing pattern, as well as increased firing frequencies of already active MUs with inspiratory and tonic (constant activity throughout the respiratory cycle) firing patterns were responsible. These data support the concept that the genioglossus is under unique motor control following arousal from sleep.

Arousal from sleep occurs upon the termination of the majority of respiratory events in obstructive sleep apnea (OSA) [1]. While, historically, arousal had been thought to play a critical role in the reopening of the airway [2, 3], it has more recently been argued that, at least for the majority of patients with OSA, its occurrence may be merely coincidental and unnecessary [1, 4]. Moreover, the question of whether arousal at event termination is beneficial, or predisposes to further respiratory events, has been under debate for many years. Arousal may be beneficial in that it is associated with a marked increase in ventilation [5], which allows the rapid restoration of normal blood-gas tensions. This is of particular relevance in situations involving large blood-gas disturbances, such as in cases of severe OSA [2, 6]. Alternatively, arousal may be viewed as being deleterious, in that it may cause a ventilatory overshoot, leading to hypocapnia and thus promoting further respiratory instability [5]. As well as destabilizing central respiratory control, the resultant hypocapnia has been thought to lead to a subsequent reduction in the activity of upper airway dilator muscles, and therefore a greater propensity toward further airway collapse. Support for this concept has been provided by studies in which hypocapnia was induced during stable sleep [7–9]. However, several studies have reported that, following arousal, the activity of the largest upper airway dilator muscle, the genioglossus, actually remains elevated above pre-arousal levels for several breaths following the return to sleep in healthy individuals [10–13] as well as in patients with OSA, both on continuous positive airway pressure (CPAP) [13, 14] and off CPAP [15, 16]. The elevated genioglossal activity is associated with increased ventilation and decreased epiglottic pressure, suggesting improved airway patency [15]. Importantly, elevated dilator muscle activity following arousal has been demonstrated to occur despite the presence of hypocapnia, which suggests that it may be the result of a mechanism operating independently to that of conventional respiratory control [15, 16]. These effects may be attributable to a slower return to sleeping levels of drive for upper airway muscle as compared to respiratory drive (with the resumption of sleep being a continuum [17]) the phenomenon of after-discharge (sometimes referred to as short-term potentiation), whereby elevations in muscular activity persist beyond the withdrawal of the initial excitatory stimulus.

Using multi-unit electromyography (EMG) recording techniques, after-discharge was first demonstrated in the genioglossus following hypoxia in healthy awake humans [18], and subsequently following obstructive events induced by transient reductions in CPAP during sleep in patients with OSA [19, 20]. The latter studies showed that genioglossal activity remained elevated beyond the return to normal levels of ventilation, which indicates that there was a degree of decoupling of the hypoglossal and phrenic motor neuron pools, and also suggests that the increased activity was the result of a mechanism operating independently of the negative pressure reflex [18–20]. Such decoupling may be an important factor in overcoming or preventing further obstruction, as a disproportionate increase in dilatory muscle activity provides the potential for improved airway patency [3]. Two studies, however, reported a reduction in the length of the after-discharge period when the events were terminated with arousal (as compared to when no arousal occurred), suggesting that arousal may have an inhibitory effect on after-discharge [19, 20]. By contrast, Cori and colleagues [21] provided evidence of genioglossal after-discharge being elicited directly by arousal in healthy subjects. The effect occurred despite the presence of hypocapnia, providing further support for its independence from changes in chemical drive [21]. What drives the genioglossus following the return to sleep after arousal is currently unknown, but single motor unit (MU) recordings of the genioglossus may provide some insight as they allow pre-motor inputs to the muscle to be investigated.

Recordings of individual MUs in the genioglossus have revealed intricate patterns of activity that suggest a complex arrangement of pre-motor inputs that is typically concealed in multi-unit recordings. Broadly, these MUs have firing patterns that either show no respiratory modulation (tonic), or that do show respiratory modulation, exhibiting peak activity during inspiration (inspiratory modulated) or peak activity during expiration (expiratory-modulated). Respiratory modulated units may either be active only during inspiration (Inspiratory Phasic (IP)) or expiration (Expiratory Phasic (EP)), or be active throughout the respiratory cycle with an increased firing rate during inspiration (Inspiratory Tonic (IT)) or expiration (Expiratory Tonic (ET)). Recently, our group demonstrated that after-discharge occurring in the genioglossus following hypoxia was predominantly due to the recruitment of previously silent inspiratory-modulated MUs, with little change in the firing rates of previously active units [22]. Similarly, Wilkinson et al.[23] reported a disproportionate increase in the activity of inspiratory-modulated units during spontaneous arousal. This increased activity was accounted for by the recruitment of new inspiratory units, an increase in the proportion of the breath for which inspiratory units were active, and also by some IP units assuming an IT pattern, whilst only a minor increase in discharge rate was noted [23]. Interestingly, expiratory-modulated and tonic units showed a complex pattern by which some units were recruited whilst others ceased firing, resulting in a minor net decrease in the activity of these units, and suggesting that a reorganization of their activity had taken place [23]. Wilkinson et al. [23] study focussed on the changes that occurred immediately upon awakening (the first three breaths following the onset of spontaneous arousal), and analyses were conducted regardless of whether those arousals resulted in full awakenings or if there was a subsequent return to sleep. As such, the nature and distribution of single MUs contributing to the observed persistence of genioglossal activity following the return to sleep after arousal remains unknown. The aim of the present study was thus to determine which MUs are responsible for the sustained increase in activity of the genioglossus on the return to sleep following brief arousal. Given the findings of previous studies, we hypothesized that this persistent activity is due to the continued firing of inspiratory-modulated MUs that are recruited during arousal.

## Methods

### Participants

Thirty-four healthy participants (13 men, 21 women, aged 19-47 years) took part in the study. Participants all reported having regular sleep patterns, good sleep quality and having not travelled across time zones or participated in shift work within the previous month. None of the participants were smokers or took any medications other than oral contraceptives, and none had difficulty breathing nasally. The University of Melbourne Human Research Ethics Committee approved the study and all participants gave written informed consent to their participation.

### Protocol

Participants individually attended the John Trinder Sleep Laboratory at the University of Melbourne in the evening approximately 2 hours prior to their usual bedtime, having consumed their evening meal at least 2 hours earlier, and having not consumed caffeine for 12 hours or alcohol for 24 hours. Participants were given an opportunity to prepare for bed before being instrumented with sleep monitoring equipment. At approximately half an hour prior to their usual bedtime, the participants lay supine on a bed and the recording electrodes were inserted into the genioglossus. They were then fitted with a nasal mask, pneumotachograph and oximetry probe for measurement of respiratory variables, as well as headphones for the delivery of auditory stimuli. Once participants were comfortable, bio-calibrations were performed, and some trial auditory tones were delivered to familiarize the participants with the different sound intensities. A 5-minute period of relaxed wakefulness was then recorded before the lights were switched off and participants were allowed to fall asleep.

Auditory stimuli (1000 Hz tones, 0.5-2s, 45-100 dB) were delivered through ear insert headphones (E-A-RTone, Cabot Safety Corporation/Auditory Systems Division, IN) during stable NREM sleep, with the intensity and duration of the stimuli being manually titrated to induce arousals. Auditory tones were delivered following at least 2 minutes of sleep that was observed to have been uninterrupted by arousal. If the stimulus did not successfully elicit an arousal, a minimum of 1 minute was allowed to pass before delivery of a louder, or if already 100 dB, a longer tone. Once at least 10 auditory-induced arousals were obtained, participants were woken, recording equipment was removed, and they were allowed to go home.

### Instrumentation

#### Sleep monitoring

For the scoring of sleep and arousals, surface electrodes were used to record central and occipital electroencephalogram (EEG) (C3 and O1, referenced to M2), left and right electrooculogram (EOG), and a bilateral masseter EMG (EMGmas). EMGmas was recorded because the positioning of the intramuscular genioglossal electrodes was such that it precluded the use of the standard AASM [24] submental EMG placement. Heart rate was monitored via surface electrodes positioned over the right clavicle and left lower rib in a modified lead II electrocardiogram (ECG) configuration.

#### Genioglossal electromyogram

Genioglossal activity was recorded from four monopolar intramuscular wire electrodes inserted percutaneously to a depth of 25 mm, at locations 10 and 20 mm posterior to the posteroinferior margin of the mandible and 5 mm lateral to the midline (each side). Each wire was inserted using a 25-gauge needle, 20-30 minutes after the application of topical local anaesthetic (Lidocaine—Prilocaine; AstraZeneca Pty Ltd, NSW, Australia) to the submental area. GG EMG signals were amplified and band-pass filtered from 30 to 3 kHz (model P511, Grass TeleFactor; Grass Technologies, West Warwick, RI).

#### Respiratory variables

Participants were fitted with a leak-proof nasal mask (Modified Profile-Lite, Phillips, Respironics, Murrysville, PA). A heated pneumotachograph (model 3700; Hans Rudolph, Shawnee, KS) was attached to measure ventilation. Mask pressure was monitored (Pmask; DP45; Validyne, Northridge, CA) through a port in the mask. Mask co_2_ and O_2_ were continuously sampled from another port in the mask (CD-3A analyser; Ametek, Berwyn, PA). Arterial oxygen saturation (SpO_2_) was measured with a pulse oximeter (Radical 7 Oximeter; Masimo, Irvine, CA, USA) sensor attached to the right earlobe.

#### Data acquisition

Data were recorded on a computer using an analogue to digital converter (1401plus and Spike2 software, Cambridge Electronic Design, Cambridge, UK). EEGs, EOGs and masseter EMG were sampled at 250 Hz and the ECG at 500 Hz. GG EMGs were sampled at 10 kHz and respiratory variables at 125 Hz.

### Data analysis

#### Sleep scoring

Studies were visually scored for sleep and arousals by an experienced scorer (AD) in accordance with the AASM criteria [24]. Scoring was done with only the EEG, EOG, and the EMGmas channels visible, so that the scorer was blinded to experimental events and changes in other variables. Close attention was paid to accurately determining the start and end times of arousal events. In addition, sub-criteria arousals were scored as events that occurred without 10 seconds of preceding sleep, but which otherwise met AASM arousal criteria.

#### Analysis of MUs

Arousals were considered suitable for analysis if they started within 5s of a tone, they were 3-15s in duration, and they were preceded and followed by > 1 min of NREM sleep (i.e. neither the epoch of the arousal, the two epochs prior nor the two epochs following, were classed as stage R or stage W). It was further necessary that no other arousals (including sub-criteria arousals scored as above) occurred in the 60s prior or the 30s after the arousal event of interest. If a secondary arousal occurred more than 30s after the arousal of interest, only breaths prior to the secondary arousal were included in the analysis. It was also necessary that there were no gross movements during the trial, and no swallow or movement occurred that would obscure any change in the firing of a single MU during the critical period for analysis (regarded as the period from three breaths before the arousal to 30s after the arousal). If a minor body movement or swallow occurred outside of this period, the trial was included but any individual breaths affected by the event were excluded from the analysis.

For a trial to be included in the analysis, it was necessary that there was at least one sortable MU present, and that the unit/s were present for > 2 breaths in order that they could be categorized in accordance with their discharge pattern. MU action potentials (spikes) were identified by Spike 2 analysis software (Cambridge Electronic Design, Cambridge, UK) on the basis of their amplitude, duration and shape using a spike triggered threshold voltage. Additional software (Spike2 script courtesy of Neuroscience Research Australia, Sydney, NSW, Australia) was then used to visually inspect and manually edit each spike where necessary, based on its amplitude, shape and frequency characteristics. The decomposition of each MU was reviewed and was either returned for further sorting or discarded if considered inaccurate. In addition to the quantification of MU discharge activity (see below section, Genioglossus MU quantification) each unit was classified according to its within-breath discharge pattern as follows.

The within-breath discharge pattern refers to the variation in a unit’s discharge rate as a function of the respiratory cycle. Five of the six within-breath discharge patterns originally described by Saboisky et al. [25] were identified in the current study: IP, IT, EP, ET, and Tonic (TT). The degree of respiratory modulation in units with a tonic component was assessed by cross-correlating the unit’s discharge rate (instantaneous frequency values) with tidal volume over a breath. As for previous studies [25], units having average cross correlation values greater than or equal to 0.4 were designated as being respiratory modulated (IT and ET units), whilst those with values less than 0.4 were designated as not being respiratory modulated (TT units). The phase of modulation for phasic units (IP versus EP) was determined by visual inspection.

#### Breath by breath measurements of MUs and respiratory activity

**Figure 1.**
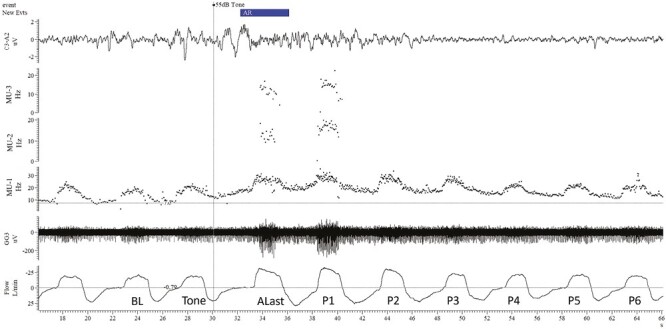
An example of motor unit recordings during and following an arousal showing the designation of breaths relative to arousal. BL = baseline, tone = breath during which the tone was played during expiration. This breath was not analyzed as a prolongation of expiration was common. Alast = the last breath of the arousal from sleep. In this case, the arousal was only a single breath in duration. P1-P6 = the first six breaths following the return to sleep. Airflow (Flow), one genioglossus recording (GG3), three instantaneous firing frequencies of individual motor units derived from the genioglossus recording shown (MU1-3), and EEG (C3-A2). Data are shown before and after a 55-dB tone was played. The time of arousal (scored blind to all data other than EEG, EOG, and masseter EMG) is indicated in the New Evts channel. Two inspiratory phasic MUs (MU-2 and MU-3) were recruited during arousal (ALast) and continue to fire only on the first post arousal breath (P1). In contrast an inspiratory tonic MU that was already active during stable sleep (MU-1) increased its firing frequency during arousal and for several post-arousal breaths.

MU firing and respiratory data were quantified on a breath-by-breath basis as follows. The breath before that during which the tone was played was designated as baseline (BL). Breaths during which the tone was played, and those between the tone and the onset of arousal, were excluded from analysis. Due to the variable length of arousals, not all arousals included more than one breath, thus only the last breath of each arousal (Alast) was included in the analysis. If the return to sleep occurred during the last half of inspiration or during expiration, that breath was designated Alast, whereas if the return to sleep occurred during the first half of inspiration, then the previous breath was designated Alast. The 10 subsequent post-arousal breaths were designated P1-P10 consecutively. An example arousal with breaths marked is shown in Figure 1.

#### Genioglossus MU quantification

The rate of discharge of MUs was quantified as for our prior studies, using software developed by Neuroscience Research Australia (Spike 2 script). Three measures were calculated for each breath across a trial: *peak frequency* (peak of a 200 ms running average during inspiration for IT, IP, and TT or expiration for ET and EP); *mean frequency* (the mean firing rate during inspiration for IT, IP, and TT or expiration for ET and EP); and *tonic frequency* (the mean firing rate over 500 ms of the non-respiratory modulated phase for units containing a tonic component). In addition, minute ventilation (V̇i), peak inspiratory flow (PIF), tidal volume (vt), breathing frequency (fb), end tidal CO_2_ partial pressure (petCO_2_), and nadir oxygen saturation (SaO_2_) were calculated for each breath.

The data underlying this article will be shared on reasonable request to the corresponding author.

### Statistical analyses

#### Respiratory variables and overall genioglossal activity

Changes in respiratory variables were assessed with a 1 (variable) × 12 (Breath: Baseline, Alast, P1-10) repeated measures analysis of variance (ANOVA). Global genioglossal activity (number of MU action potentials per breath) and the contribution of the different unit types was assessed with a 12 (breath, as above) × 5 (unit types) ANOVA with *p* < .05 considered significant. In order to explore significant interaction effects, 1 × 12 ANOVAs were subsequently performed for each MU type. In addition, to ensure that data from individual subjects did not contribute substantially to the group results, the 12 × 5 ANOVA was repeated using subjects rather than individual MUs as the unit of analysis.

#### Mechanism of contribution of different MU types

To enable the assessment of recruitment/de-recruitment, the distribution of the different MU discharge patterns was tabulated for each breath following arousal, including changes from one discharge pattern to another following the arousal. Differences in the distributions (as compared to baseline) were tested using the χ² test. To enable the assessment of rate coding (firing frequency changes) in already active MUs, additional 1 × 12 repeated measures ANOVAs were performed separately for the three measures of MU firing (peak, mean, and tonic activity) for each MU type. Given that there were 12 ANOVAs performed (four MU types (IP, IT, ET, and TT [not enough EP units were present for analysis]) and three frequency measures, a probability of < .004 was considered significant for these comparisons (Bonferroni correction of 0.05/12). Greenhouse Geiser corrected *p* values are reported throughout due to asphericity in much of the data.

## Results

Of the 34 participants in the study, one participant did not attain stable sleep and therefore all data from this individual were excluded. A total of 1089 auditory tones were played to the remaining participants with a mean of 32 tones per participant (range 1-95). For five participants there were no arousals that met criteria for inclusion in the analysis, and for a further 8 there were no MUs present during or following arousal. Data for the remaining 20 participants were included. These 20 participants (13 female) were aged 23 (4.2) years, had a mean BMI of 22.5 (2.2) kg/m^2^, and had 323 induced arousals. Of these, 98 arousals had sufficient data for inclusion in accordance with the abovementioned criteria, which yielded a total of 239 sorted MUs.

### Respiratory activity

**Figure 2.**
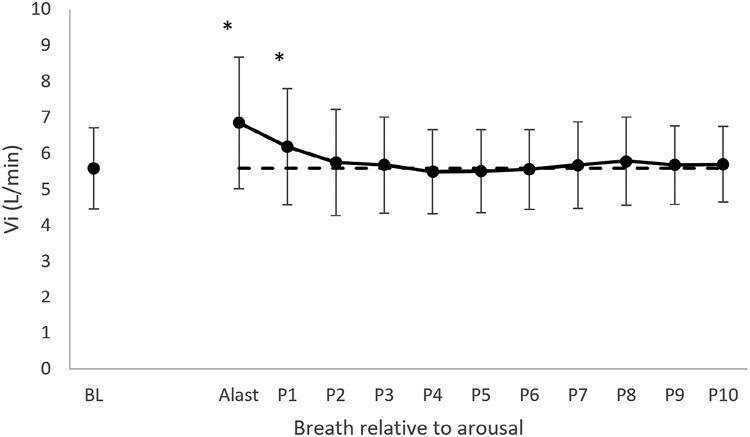
Minute ventilation (V_I_) on the last breath prior to the auditory tone (BL), last breath of arousal (ALast) and first 10 breaths following the return to sleep (P1-P10) in 98 arousals from 20 healthy young individuals in whom MU data were obtained. *p < .001 increased from BL. Mean and SD are shown. The dotted line indicates the baseline ventilation level.

Minute ventilation (V̇i; Figure 2) increased during arousal and slowly declined (significant breath effect *p* < .001) with the last breath of arousal (Alast) and P1 being significantly elevated above BL. Tidal volume (*p* < .001) and breathing frequency (*p* < .001) also showed significant increases on ALast and P1 compared to BL. PIF was elevated above BL for ALast, P1 and P2 (*p* < .001). petCO_2_ was slightly (~1 mmHg) lower than BL on P1 and P2 (*p* < 0.001).

### Genioglossal activity

**Figure 3.**
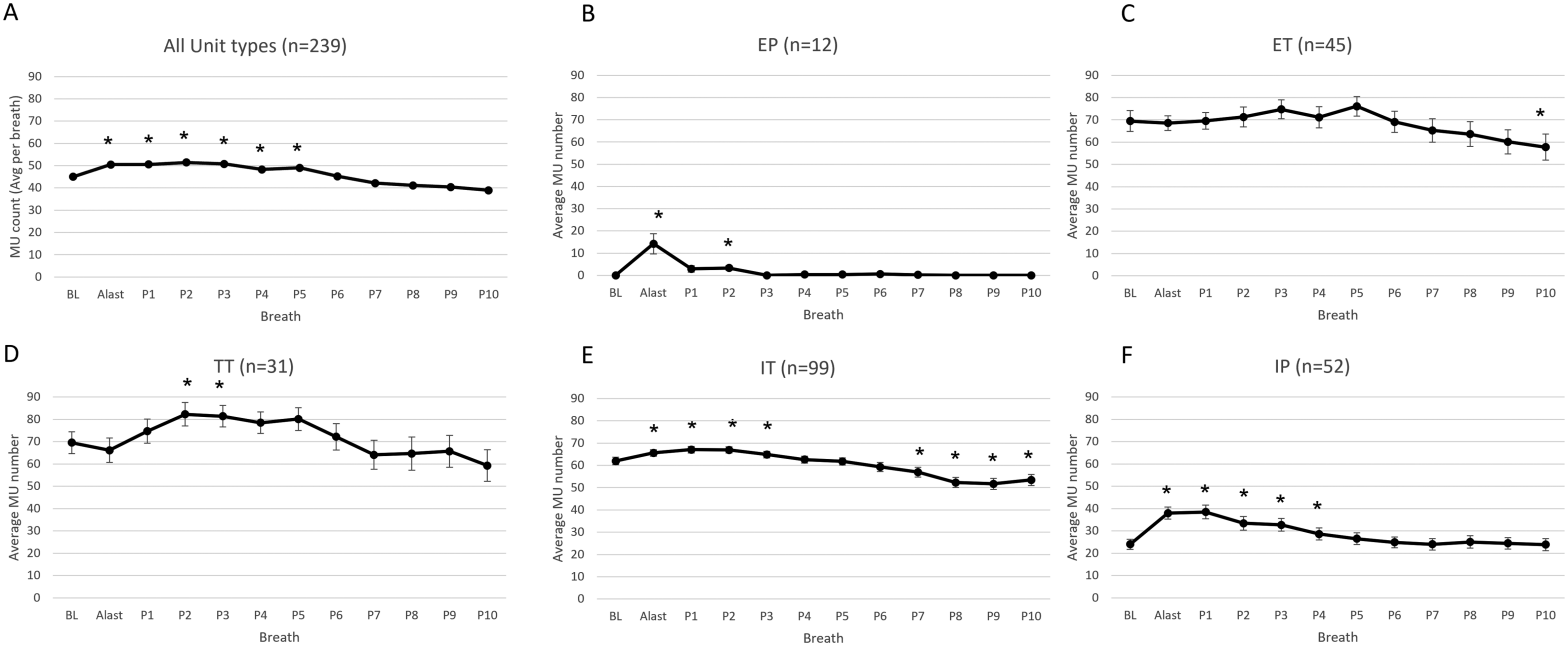
The average number of MUs per breath across all unit types on the last breath prior to the auditory tone (BL), last breath of arousal (ALast), and first 10 breaths following the return to sleep (P1-P10) in 20 healthy young individuals. *p < .05 increased from BL on post hoc testing. Mean and SD are shown. IP = inspiratory phasic, IT = inspiratory tonic, EP = expiratory phasic, ET = expiratory tonic, TT = tonic. *n* represents the number of MUs averaged in each panel.

A total of 208 MUs were present at baseline (in 18 participants) during stable sleep. The distribution of the different within-breath patterns is shown in line 1 of Table 1. The average number of MU action potentials per breath was elevated above baseline (Figure 3A, ANOVA main effect for Breath *p* < .001) during the arousal and for five breaths following the return to sleep (P1-P5 higher than BL) indicating after-discharge consistent with that observed in multiunit studies. There was also a significant main effect of MU type (*p* = .001, EP and IP having fewer MU action potentials per breath than units with a Tonic component) as well as Breath by Type interaction effect (*p* < .001). Of note, secondary arousals occurred between P5 and P10 in 18 trials affecting 25 MUs resulting in the apparent slight reduction in overall activity beyond P5.

**Table 1.** The number and proportion of motor units with different firing patterns present on breaths before during and following brief arousal from sleep

Baseline %	IP	IT	EP	ET	TT	Total
	42 (*N* = 6) 20.2	98 (*N* = 10) 47.1	0 0	40 (*N* = 8) 19.2	28 (*N* = 9) 13.5	208 (*N* = 18)
De-recruited on ALast	1			1	1	3
Recruited on ALast	9	1	10	4	1	25
Converted to a different pattern by ALast	16	2	0	0	0	
Converted to this pattern by ALast	2	16	0	0	0	
**Total MUs at ALast** **%** χ² **= 1.30, *p* = .73**	**36** **15.7**	**113** **49.1**	**10** **4.3**	**43** **18.7**	**28** **12.2**	**230 (*N* = 20)**
De-recruited on P1	1		5	1		7
Recruited on P1				1	2	3
Converted to a different pattern by P1	6	5			1	
Converted to this pattern by P1	5	6		1		
**Total MUs on P1** **%** χ²**=1.86; *p* = .603**	**34** **15.0**	**114** **50.4**	**5** **2.2**	**44** **19.5**	**29** **12.8**	**226 (*N* = 19)**
De-recruited on P3	4		5	2		10
Recruited on P3	1					1
Converted to a different pattern by P3		2		1		
Converted to this pattern by P3	2				1	
**Total MUs on P3** **%** χ²**=1.94; *p* = .585**	**33** **15.3**	**112** **51.9**	**0** **0**	**41** **19.0**	**30** **13.9**	**216 (*N* = 19)**
De-recruited on P5	3				1	4
Recruited on P5	1		1			2
Converted to a different pattern by P5		3				
Converted to this pattern by P5	3					
**Total MUs on P5** **%** χ² **= 1.408; *p* = .704**	**34** **15.9**	**109** **50.9**	**1** **0.5**	**41** **19.2**	**29** **13.6**	**214 (*N* = 18)**
De-recruited on P8	3	9	1	6	5	24
Converted to a different pattern by P8		3				
Converted to this pattern by P8	3					
**Total MUs on P8** **%** χ²**=0.64; *p* = .887**	**34** **17.9**	**97** **51.1**	**0** **0**	**35** **18.4**	**24** **12.6**	**190 (*N* = 18)**

*N* = number of participants contributing to the data. χ² show differences between the particular breath during or after arousal and baseline (prior to arousal). ALast = last breath of the arousal. P1, P3, P5, and P8 = the 1st, 3rd 5th, and 8th breath following the return to sleep after arousal.

The bold values refer to the total number (and %) of motor units that fired on a given breath.

Post hoc testing on the number of MUs active on each breath for the individual MU types indicated that significant changes over breaths occurred for all unit types (EP *p* = .009, ET *p* = .009, TT *p* = .008, IT *p* < .001, IP *p* < .001). However, the breaths and direction of change differed for the different unit types. The number of EP units (Figure 3B) was elevated above baseline during the arousal and breath P2 only, whereas the number of ET units (Figure 3C) was unchanged until the last post arousal breath where they fell below baseline. The number of TT units (Figure 3D) was higher than baseline only on breaths P2 and P3. In contrast, the number of IT units (Figure 3E) was higher than baseline during arousal and on breaths P1-P3, but then fell below baseline on breaths P7-P10. On the other hand, the number of IP units (Figure 3F) increased during arousal and remained elevated for breaths P1-P4 but did not fall below the baseline level.

In order to assess whether results were biased by individual participants contributing a high proportion of a particular MU type, the 12 × 5 ANOVA was repeated with participant as the unit of analysis. In this analysis the main effects of breath (*p* = .005) and MU type (*p* < .001) remained, but the breath by MU type interaction was no longer significant (*p = *.636), potentially due to reduced statistical power.

### Recruitment/de-recruitment during arousal and on the return to sleep

Three MUs ceased firing during arousal, whereas 31 new MUs were recruited during arousal and the post-arousal breaths (Table 1). Although 12 EP units were recruited during arousal, 4 fired only during the arousal period, 4 stopped firing on the second post arousal breath (P2) and the remaining 4 were intermittently active firing for 1-3 breaths over P3-P7. In total, EP units only contributed an additional 95 individual MU action potentials (out of a total 4306 recruited MU action potentials) in the entire post-arousal period. Likewise, of the five recruited ET units, one only fired during arousal, one ceased firing on the first post arousal breath, with the remaining 3 units continuing to fire until after P7. Of the 10 recruited IP units, 3 ceased firing after the first post arousal breath (P1), one ceased firing after breath P2, whereas all others continued firing for at least 3 of the remaining 7 breaths, contributing a total of 711 additional MU action potentials in the post arousal period. The single IT unit that was recruited continued firing until breath P6 and the 3 TT units that were recruited during arousal were active the entire recovery period. Thus, the increased genioglossal activity following return to sleep after arousal was (at least in part) due to persistent firing of previously silent MUs that were recruited during arousal. The number of MUs active at baseline, the last breath of arousal, P1, P3, P5, and P8 is shown in Table 1.

### Changes in unit firing patterns (conversion between unit types)

Sixteen units that fired with an IP pattern at baseline developed a tonic component and therefore became IT units following arousal. All except two of these units gradually resumed an IP firing pattern over the post-arousal period (shown in Table 1 as “converted to” IP units). Following arousal, two units that fired with an IT pattern lost their tonic component and therefore assumed an IP pattern. One of these converted back to an IT pattern at breath P6 and the other continued firing as an IP unit for the post arousal period. One of the recruited EP units developed a tonic component for a single breath on P1 before ceasing to fire. One TT unit converted to an ET pattern during arousal and for the first 2 post arousal breaths before resuming a TT firing pattern. ET units were not observed to change firing pattern during arousal or on the return to sleep. Overall, the distribution of unit patterns did not differ from baseline to any breath post arousal (Chi squared test results in Table 1, *p* > .05 for all comparisons).

### Changes in firing frequency of units active prior to arousal

The peak firing frequency of already active ET units did not change during arousal or the return to sleep (ANOVA breath effect *p* = .027). In contrast, TT, IT, and IP peak firing frequencies all changed over time (*p* = .001, <.001, and < .001 respectively). TT units were not altered during arousal, but fired at a higher frequency for all post arousal breaths, whereas IT frequency was above baseline during arousal and until P3, and IP units fired at a higher frequency during arousal and until P5.

**Figure 4.**
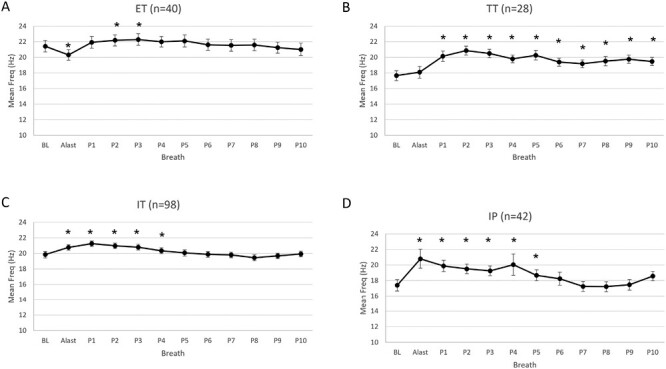
Mean firing frequency changes of already active units. The mean firing frequency of MUs on the last breath prior to the auditory tone (BL), last breath of arousal (ALast), and first 10 breaths following the return to sleep (P1-P10) in 20 participants. *p < .05 increased from BL on post hoc testing. Mean and SD are shown. IP = inspiratory phasic, IT = inspiratory tonic, ET = expiratory tonic, TT = tonic.

The mean firing frequencies of all units types changed significantly over arousal and the return to sleep (ANOVA Breath effect *p* < .004 for all unit types, Figure 4). For ET units, mean firing frequency decreased during arousal before returning to the baseline level and then slightly increasing above baseline for breath P2 and P3. For TT units mean firing frequency did not change during the arousal but was elevated above baseline for the entire post arousal period. In contrast, the mean firing rate of IT and IP units increased during arousal and remained elevated above the baseline level until P4 and P5, respectively.

The tonic firing frequency of ET, TT and IT units also all changed significantly over the arousal and return to sleep (ANOVA breath effect *p* < .001 for all unit types). For ET units firing frequency again tended to be reduced during the arousal but was slightly higher than baseline during breaths P2-P5. For TT units, firing frequency was unchanged during arousal and P1, but higher than baseline on breaths P2-P6. For IT units firing frequency was higher than baseline both during the arousal and all breaths until P5. Although the changes in firing frequency reached statistical significance, it should be noted the mean firing frequency only changed 1-3 Hz following arousal and on the return to sleep.

## Discussion

The results of the present study indicate that the sustained increase in genioglossal activity on the return to sleep following transient arousal is predominantly due to increased activity of MUs with an IP and IT pattern. However, contrary to our hypothesis, this increased activity was not only a result of persistent firing of newly recruited units, rather it was also a result of conversion of IP to IT units as well as increased firing rates of IP and IT units that were already active prior to the arousal. Further, also in contrast to our hypothesis, units with a TT pattern were found to have increased firing rates during the post-arousal period, despite not being elevated during the arousal itself. Although some units with an EP and ET pattern were recruited at arousal, their firing was intermittent and short lived, and for the ET units occurred concurrent with a reduction in firing frequency of already active units (no EP units were active at baseline) suggesting these units did not contribute importantly to the persistent genioglossal activation observed following the return to sleep after arousal.

These results are broadly consistent with previous findings from our laboratory showing the abrupt increase of genioglossal activity immediately following arousal is largely due to the recruitment of inspiratory-modulated units [23]. However, it should be noted that whereas significant changes in the firing frequency (rate coding) of inspiratory MUs were also observed following arousal in the current study, this effect was smaller and not always statistically significant in our prior study investigating MUs immediately following arousal [23]. In addition, the recruitment of new units at arousal was less marked in the current study when compared with the prior study [23]. Thus, at the end of a transient arousal and during the return to sleep, the elevated genioglossal activity appears to result from a combination of a slightly higher firing rate in the already active tonic and inspiratory MUs, recruitment of new inspiratory MUs and changes in firing patterns of some units from IP to IT.

**Figure 5.**
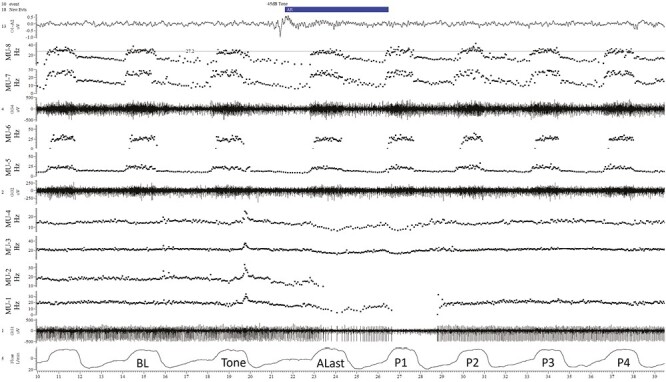
An example of inhibition of tonic units concurrent with minimally changed IP unit firing rates. BL = baseline, tone = breath during which the tone was played during expiration. This breath was not analyzed as a prolongation of expiration was common. Alast = the last breath of the arousal from sleep. In this case, the arousal was only a single breath in duration. P1-P4 = the first 4 breaths following the return to sleep. Airflow (Flow), three genioglossus recordings (GG1, GG2, and GG4), eight instantaneous firing frequencies of individual motor units derived from the genioglossus recording shown (MU1-8) and EEG (O1-A2). Data are shown before and after a 45 dB tone was played. The time of arousal (scored blind to all data other than EEG, EOG, and masseter EMG) is indicated in the New Evts channel.

An additional similarity between the current study and our prior study [23] is the variable response that MUs with tonic and expiratory patterns demonstrated at arousal from sleep. While some TT and ET units were recruited or increased activity with arousal, there were also examples of inhibition of these units during arousal (see Figure 5 for an example). The proportion of ET units that increased was approximately offset by those that were decreased leading to no overall change in activity during arousal, whereas firing of IP and IT units were near universally increased or unchanged (not decreased) during arousal. This further supports the concept that the control of tonic and expiratory active MUs is distinct from, and more complex than the control of inspiratory-modulated units [26].

Horner et al. [27] proposed that there exists upon awakening a transient arousal state that is neurophysiologically distinct to prolonged wakefulness. In a meticulous study conducted on rats, the group demonstrated a reduction in sensorimotor gating immediately following awakening as compared to later wakefulness. This was taken to indicate an enhanced state of central nervous system responsiveness to external stimuli that is likely to fulfil a biological imperative of preparedness for immediate response to threat during this period. The results of the present study are consistent with this model, in that arousal may activate a state of preparedness for high respiratory drive that exceeds the physiological demands of wakefulness per se, and the persistent effects of which override the typical state-dependent changes that occur during the subsequent transition back to sleep. However, our results suggest that this neurophysiologically distinct arousal state appears to only activate inspiratory-modulated MUs, with expiratory and tonic units showing variable responses including inhibition.

If such a transient arousal state is activated in humans, it may slowly dissipate following the return to sleep, or alternatively, the return to sleep itself may be a gradual process [17]. Another possibility is that after-discharge of genioglossal activity may occur following cessation of the transient state and give rise to the slow decline of genioglossal activity following the return to sleep after arousal. Arousal has been shown in two studies to lower the magnitude of genioglossal after-discharge elicited by obstructive respiratory stimuli [19, 20]. Further, the individual MUs responsible for after-discharge following hypoxia appear almost universally to be a result of continued firing of recruited IP and IT units, with minimal input from TT or expiratory units [22], which is in contrast to what was observed in the current study during the return to sleep following arousal, where increased frequency of TT units was also observed. This raises the possibility that the elevated genioglossal activity following the return to sleep after arousal may be a result of a slowly dissipating transient arousal state rather than after-discharge per se. Ultimately, further research will be required to determine the mechanism of prolonged genioglossus activation following the return to sleep after arousal.

### Proposed model of genioglossus motor control

Taken together, the observations of MU activity in this and past studies have indicated that most of the variations in the activity of the genioglossus that occur in response to alterations in respiratory drive can be attributed to changes in activity of inspiratory-modulated units. This has been demonstrated to be the case with: sleep–wake state-dependent changes invoking the withdrawal of the wakefulness stimulus at sleep onset [28], and its reinstitution upon arousal from sleep [23]; chemical stimulation, including hypercapnia [29], and hypoxia [22]; and, mechanical stimulation such as upper airway resistive loading [30]. Contrasting with these gross changes in inspiratory-modulated units, MUs with tonic and expiratory patterns have shown a more complex response in which some units alter their firing pattern, and some units are recruited whilst others are de-recruited [26]. On the basis of these observations, we have proposed a model whereby innervation of the genioglossus from the hypoglossal motor nucleus occurs via two relatively discreet pathways, with an “inspiratory system” that aims to maintain airway patency by modifying overall activity levels to compensate for the magnitude of the respiratory load, and a “tonic system” that optimizes the shape of the airway in response to more localized demands [26]. The findings of the present study are consistent with this model, in that we would expect, as our previous results have shown, that the reintroduction of the wakefulness stimulus upon arousal would result in an increase in respiratory drive and activation of inspiratory-modulated units. However, this activation persists beyond the subsequent transition back to sleep. Interestingly, the activation of tonic MUs observed in this study only occurred as respiratory drive returned to baseline levels, again consistent with something other than respiratory drive (such as a transient arousal state) activating these MUs.

### Clinical implications

Whether transient arousal is beneficial or detrimental in OSA has been in debate for many years. This study has added to those demonstrating that arousal results in a persistent elevation of genioglossal activity that continues following the return to sleep. We further demonstrated that this is largely related to activation of inspiratory modulated MUs. We have previously noted with multi-unit recordings that genioglossus activity progressively increases over successive respiratory events (with arousal) in OSA and that following this rise stable breathing/slow wave sleep often occurs [13, 16]. Thus, there would appear to be the theoretical potential for arousals (and the resulting slow decay in genioglossal activity) to be utilized as a means by which to harness elevations in activity of upper airway dilator muscles in the treatment of OSA. Of course, for such a treatment to be effective it would be necessary that any gain in upper airway stabilization achieved by increases in genioglossal activity is sufficient to compensate for the likely destabilization of central respiratory control caused by the hyperventilation that also occurs in response to arousal. Furthermore, it would remain critical that there was a net reduction in the number of arousals, so as not to augment the known adverse health consequences of arousal related to the associated cardiovascular activation and sleep fragmentation. However, utilizing small arousals or harnessing their neurobiological underpinnings with pharmacotherapy or other means would have the advantage of targeting of the appropriate “compartment” of the hypoglossal motor nucleus (inspiratory-modulated MUs). Clearly, more research is required to determine whether harnessing the dilator muscle stimulatory effects of arousal would be viable in OSA treatment.

### Limitations

A potential limitation of this study is that arousals induced with external auditory stimuli may elicit a different response to that occurring during spontaneous arousal from sleep. Although it would seem unlikely given the similarities of our results with previous observations of genioglossal MUs immediately following spontaneous arousal [23], it remains possible that activity in these units follows a different course upon the return to sleep. On the other hand, it is also possible that auditory arousals are more representative of the arousals associated with respiratory events in OSA, in that the latter may also be regarded as being induced by extrinsic factors. However, the presence of both blood-gas disturbances and the reopening of the airway occurring with arousal on the termination of respiratory events may cause a different response to the isolated tone induced arousals studied here. Certainly, ventilatory responses to arousal are augmented with prior elevation in airway resistance [10, 31]. Finally, chronic untreated OSA may further alter arousal responses from those observed in this study. Clarification of these possibilities requires further investigation in OSA patients.

A further limitation for all studies assessing individual MUs is that there may be a methodological bias towards a greater representation of units that were active during milder muscle responses because individual MUs become impossible to differentiate with large muscle responses. Also, Taranto-Montemurro [20] demonstrated that after-discharge differed between stage N2 and slow wave sleep and therefore the responses observed in the current study (in which 77 of the 98 arousals occurred during stage N2 sleep), may have differed if only slow wave sleep was investigated. Finally, as some participants contributed to only one or two particular MU types, it is possible that individual differences contributed to some of the differences ascribed to MU types. Participant numbers were too small to replicate all statistical analyses averaged within participants and particular MU types, however the global changes were similar when averaged by participant. In addition, a linear mixed model performed in R (version 4.2.2, lme4 package) showed that the peak firing frequency differed over breaths and with MU type after accounting for participant and electrode site (as random effects).

## Conclusion

In conclusion, the present study demonstrated that the prolonged activation in the genioglossus upon the return to sleep following transient arousal is due to the persistent firing of inspiratory modulated units recruited during the arousal as well as an increase in firing frequency of previously active inspiratory-modulated and tonic MUs. These findings, along with those of prior studies, do not support the theory of arousal having a deleterious role in contributing to the pathogenesis of OSA via a reduction in upper airway dilator muscle activity. In fact, therapeutic strategies that harness this prolonged activity may be beneficial in the treatment of OSA.
